# First Contact to Odors: Our Current Knowledge about Odorant Receptors

**DOI:** 10.3390/s8106303

**Published:** 2008-10-09

**Authors:** Hyoung-Gon Song, Jae Young Kwon, Hyung Soo Han, Yong-Chul Bae, Cheil Moon

**Affiliations:** 1 Department of Emergency Medicine, Samsung Medical Center, Sungkyunkwan University School of Medicine, Seoul, Republic of Korea; 2 Department of Biological Science, Sungkyunkwan University, Suwon, Republic of Korea; 3 Department of Physiology, School of Medicine, Kyungpook National University, Daegu, Republic of Korea; 4 Department of Oral Anatomy and Neurobiology, School of Dentistry, Kyungpook National University, Daegu, Republic of Korea; 5 Brain Science & Engineering Institute, Kyungpook National University, Daegu, Republic of Korea

**Keywords:** Odorant receptor, chemical senses, olfaction, olfactory sensory neuron

## Abstract

Chemical senses – especially smell – are known to be important for the fundamental life events such as sensing predators, selecting mates, as well as finding food. The chemical senses are decoded in the olfactory system which is able to detect and differentiate thousands of odorous substances comprised of chemically divergent structures (i.e. odorants). The high selectivity of the olfactory system is heavily dependent on the receptors for each odorants (i.e. odorant receptors). Thus, studying odorant receptors may not only facilitate our understanding the initial events of olfaction but provide crucial knowledge for developing a novel, odorant receptor-based biosensor for chemical screening. Here we provide a review of recent advances in our understanding of odorant receptors.

## 1. Introduction

Olfaction is the crucial sense for the fundamental life events such as searching for food, finding mates and avoiding predators. The olfactory system detects environmental chemicals with a member of a seven-transmembrane receptor family called odorant receptors. Studying odorant receptors may not only facilitate our understanding the initial events of olfaction but provide crucial knowledge for developing a novel, odorant receptor-based biosensor for chemical screening. Odorant receptors indeed retain their specificity and selectivity towards either known or unknown chemicals, and could be probed with an array of chemicals as a biosensor [1]. The life-long efforts searching for the odorant receptor gene family by Buck and Axel were rewarded with the Novel prize in Physiology or Medicine in 2004 [2]. Since then, scientific exploration for the odorant receptors has been accelerated, yet its potential for technological uses has just been dawning. To awake the full potential of odorant receptors for the use as a biosensor, we will here cover basic structure of the olfactory system of mammals and insects and current knowledge about their odorant receptors.

## 2. Odorant Receptors in Mammals

### 2.1. Anatomy of the olfactory system

**Figure 1.**
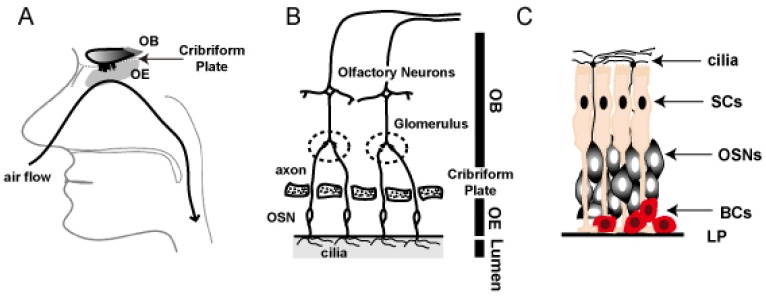
Anatomy of the olfactory system.

In most vertebrates the olfactory system is incorporated into the respiratory system in the nasal cavity. The respiratory epithelium resides in the most anterior parts of the nasal cavity, and the olfactory epithelium is located in deeper parts of the nasal cavity (Figure 1).

The olfactory bulb (OB) is divided from the olfactory epithelium by an ethmoid bone called cribriform plate (Figure 1A). Olfactory sensory neurons (OSNs) are projecting their axon bundles to the olfactory bulb (Figure 1B). Besides the main olfactory epithelium, another chemosensory organ called vomeronasal organ (VNO) exists, which plays a critical role in social and/or reproductive behavior. VNO is located within the anterior ventral end of the nasal septum. Different from the OSNs in the olfactory epithelium, the vomeronasal sensory neurons in the VNO sensory epithelium are projecting their axons to the accessory olfactory bulb (AOB) residing in the posterior part of the olfactory bulb.

Three distinct cell types exist in the olfactory epithelium; OSNs, sustentacular cells (SCs), and basal cells (BCs) (Figure 1C). OSNs comprise 75-80% of the cells in the olfactory epithelium [3]. OSNs are bipolar neurons, extending apical dendrites to the surface of the neuroepithelium and sending unmyelinated axons to glomeruli in the olfactory bulb of the brain. And OSNs are the only neurons that form a direct conduit between external chemical environment and the brain. The apical dendrites form dendritic knobs from which arise specialized, non-motile cilia, where the odorant receptors exist [4-6]. Sustentacular cells stretch from the epithelial surface to the basal lamina, where they maintain foot processes [5, 7]. Sustentacular cells electrically isolate OSNs, secrete components into the mucus, and contain detoxifying enzymes [8]. Basal cells underlie the OSNs and serve as precursors for the generation of new OSNs throughout adulthood [9-11].

### 2.2. Olfactory signal transduction

**Figure 2.**
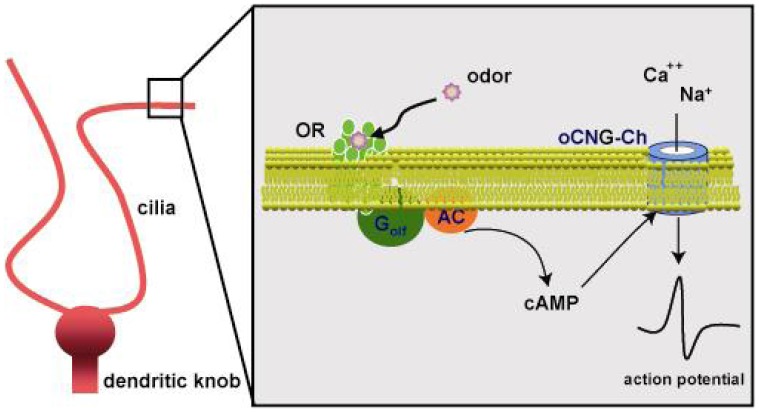
Olfactory signal transduction.

Olfactory signal transduction is initiated when odorous chemicals interact with specific odorant receptors in cilia of OSNs (Figure 2) [12-15]. Receptors subsequently couple to a G-protein to activate adenylyl cyclase (ACIII) to produce cAMP [16-18]. Cyclic AMP levels increase, and open a cyclic nucleotide-gated channel, resulting in an influx of Na^+^ and Ca^2+^ [19, 20]. The immediate response is the generation of a graded receptor potential mediated by Ca^2+^-dependent Cl^-^ channels [21, 22].

### 2.3. Odorant receptors

Initial efforts searching for odorant receptors focused mainly on finding a specific protein for a specific odorant. In 1982, Pelosi *et al.* identified a protein specifically recognizing a bell-pepper odorant, 2-isobutyl-3-methoxypyrazine (IBMP) in cow nasal turbinate [23]. Snyder's group also identified the odorant binding protein specific for IBMP from bovine and rat olfactory epithelium [24]. Further biochemical studies lead to the assumption that odorant signal transduction involved G proteins. Thus numerous researchers started to search for Ors from G protein-coupled receptor families, and Buck and Axel finally identified a very large gene family of closely related olfactory-specific seven transmembrane spanning domain receptors by polymerase chain reaction (PCR) [25]. Since then, numerous odorant receptor genes have been isolated from 12 vertebrate species: rat, mouse, human, catfish, zebrafish, dog, frog, chicken, pig, opossum, mud puppy and lamprey [26]. Odorant receptors may be categorized into two groups, Class I (fish-like) and Class II (tetrapod-specific) odorant receptors. Class I odorant receptors are specific for recognizing water soluble odorants, whereas Class II odorant receptors bind airborne odorants [27]. Expression of Class I odorant receptors has already been reported in rats [28] and in human [29]. Class II families which can further be classified into 19 phylogenetic clades are all present in more than one chromosome each, except for the very small family 12 [27]. In humans, the size of the receptor family genes is estimated at the range from 500 to 1,000 [30]. Such large number of odorant receptor genes implies that the first steps of odorant recognition may be accomplished within the primary sensory neurons themselves. Thus odorant receptor seems to be excellent for sensors differentiating numerous chemicals, although only one third of these genes are functional [31].

### 2.4. Expression of odorant receptors in the olfactory epithelium

Expression of odorant receptors in the olfactory epithelium demonstrates an unusual spatial distribution [32, 33]. In situ hybridization studies show that mRNAs for odorant receptors are expressed within one of several broad, non-overlapping zones. Within a zone which occupies about a quarter of the olfactory epithelium, odorant receptors are expressed in a random manner [32]. Recent observations, however, of overlapping zones in the olfactory epithelium [34-36] imply that our understanding of expression of odorant receptors in the olfactory epithelium is far beyond complete.

Most studies have been done on the expression and distribution of odorant receptors at the message level, however little information is available at the protein level. Using polyclonal antibodies raised against some odorant receptors, expression of an odorant receptor in rats is visualized as early as E14 in a zonally restricted pattern [37]. The expression of odorant receptors is restricted mostly to the cilia and dendritic knobs of OSNs. The cilia-specific expression of odorant receptors supports a primary role for odorant receptors in the olfactory transduction [38-40].

### 2.5. Heterologous expression of odorant receptors

There has been little knowledge about the ligand specificity of individual odorant receptors in any species, due to difficulties in expressing odorant receptors in heterologous systems. The primary role of odorant receptors is certainly to detect environmental odorous chemicals. However, difficulties of heterologous expression of odorant receptors severely limited studies for functional confirmation of such role. The most convincing observation concerning function were initially reported by genetic studies in C. elegans, which demonstrated that a mutant lacking odorant receptors lost its ability to detect a specific odor [41]. Krautwurst and Reed [42] firstly achieved functional heterologous expression of odorant receptors using HEK-293 cells. This group generated an expression library of mouse odorant receptors, and identified three odorant receptors responding specifically to carvone, (-) citronellal, and limonene at micromolar concentrations, respectively. Firestein and colleagues also successfully demonstrated in vivo functional expression of a rat odorant receptor clone in the nasal epithelium using a recombinant adenovirus containing a putative odorant receptor [43]. The approaches developing heterologous functional expression systems for odorant receptors facilitate screening odorant receptors at a large scale as well as developing odorant receptor mimicking biosensors.

### 2.6. Pheromone receptors

**Figure 3.**
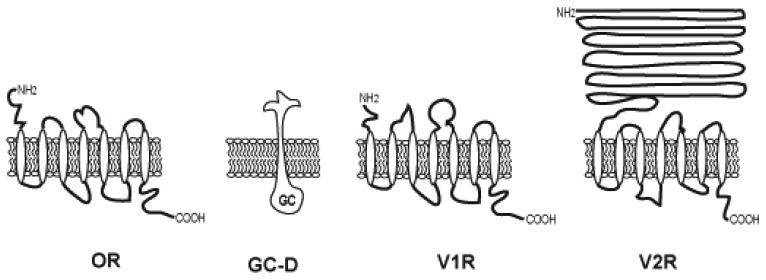
Various odorant receptors.

Vomeronasal organ is another chemosensory system located at the base of the nasal cavity. Different from the olfactory sensory organ, the VNO perceives and processes stimuli related to social and reproductive behavior (e.g. pheromones) in many species of vertebrates [44], implying that distinct families of receptors are expressed in the VNO sensory epithelium. Two families of VN receptor genes encoding proteins with seven transmembrane domains have been identified in the VNO, and indeed do not share homology to odorant receptors [45] (Figure 3). The first gene family (V1R) is expressed in apically situated receptor neurons, those co-expressing G_i2_-proteins [45]. The second gene family (V2R) is expressed in more basally situated receptor neurons that co-express G_o_-proteins [46-48]. This V2R family of genes consists of many pseudo-genes which may not code for functional receptors or orphan receptors which ligand(s) are not yet identified. The presence of at least two families of putative receptor genes adds credence to the idea that the VNS is heterogeneous and likely to respond to different stimuli.

### 2.7. Atypical odorant receptors

Some OSNs devoid of crucial signaling mechanisms for olfactory signal transduction such as G_olf_, ACIII, etc [49] express guanylyl cyclase-D (GC-D), phosphodiesterase 2 (PDE2) and cGMP-selective channel [49, 50]. GC-D is phylogeneticaly a kin to retinal Ca^2+^-regulated GC-E and GC-F which are activated by peptide ligands [51]. In particular, GC-D and GC-E/F share characteristic sequence similarity in a regulatory domain that is involved in binding of GC activating proteins [52]. This similarity raises the intriguing possibility that GC-D may play an odorant receptor role [53]. These OSNs project their axons in glomeruli different from cAMP producing OSNs.

### 2.8. Combinational odorant receptor coding for odors

**Figure 4.**
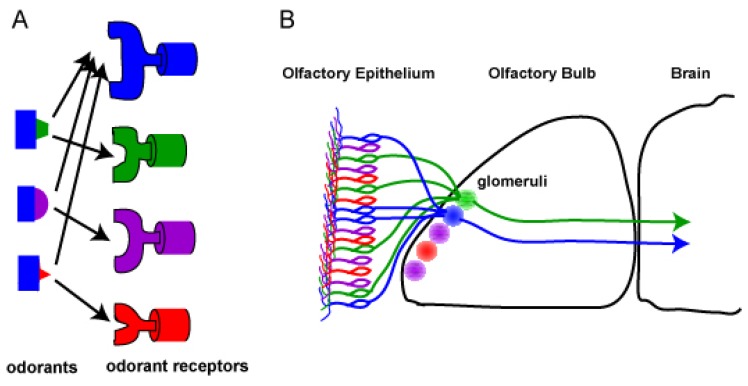
Combinational odorant receptor coding for odors.

Each odorant receptor seems to recognize multiple odorants [14], implying that the mammalian olfactory system may encode odor identity and differentiate odors via a combinational odorant receptor coding. That is, each odorant receptor serve as one component of the unique combinational odorant receptor coding, in parallel different combinations of odorant receptors are able to encode distinct odors (Figure 4A). This combinational odorant receptor coding scheme permits the differentiation of a great number of diverse odors with relatively small number of odorant receptors. For example, one billion of odorants can be differentiated only if assuming that three odorant receptors encode each odorant. To date, very limited information about odorant receptors and their ligands is available, but the list has been expanding due to the modern molecular biological techniques and imaging technology (see ref for lists [54, 55]).

Besides functioning in the detection of odorants, odorant receptors appear to be involved in determining or guiding OSN axonal projections to the olfactory bulb, and probably to specific glomeruli [56, 57]. In rodents, the axons of OSNs expressing the same odorant receptors converge onto defined glomeruli in the olfactory bulb, suggesting that the rodent olfactory bulb is topographically organized, and in turn that OSN expressing a specific odorant receptor projects to and forms a synapse with the representing glomeruli in the olfactory bulb (Figure 4B). This type of organizational wiring is also implicated in encoding odors; that is an odor is encoded by activation of a specific set of glomeruli. Taken together, the combinational odorant receptor coding scheme and the combinational glomeruli activation scheme would be greatly appreciated when the odorant receptor based biosensors would be designed.

## 3. Odorant receptors in insects

### 3.1. Odorant receptors and olfactory system in Drosophila

The fruit fly, *Drosophila melanogaster*, is an ideal model system for the study of odor coding at a molecular and cellular level, due to similarity of the process of sensing and responding to chemicals to that of higher organisms such as mammals. A remarkable advance to elucidate the olfactory system in *Drosophila* was achieved with the identification of odorant receptor (*Or*) genes [58-60]. Since then, there has been a large amount of progress in understanding the function of odorant receptors and olfactory system in this insect. Moreover, insect odorant (and gustatory) receptors have no homology to vertebrate odorant receptors, suggesting that the insect odorant receptors may detect distinct category of odors from vertebrates. This would be critical for developing odorant receptor based biosensors.

### 3.2. Olfactory organs in Drosophila

**Figure 5.**
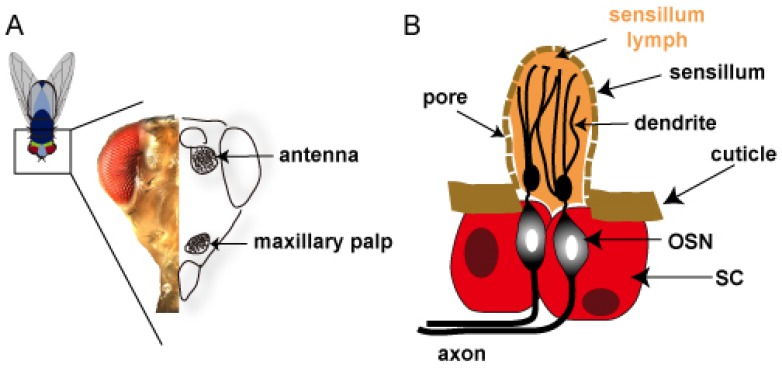
Anatomy of the insect olfactory system.

Adult flies are sensitive to a diverse range of odorants, and they have two bilaterally symmetrical pairs of olfactory organs, the third segment of the antennae and the maxillary palps, located on the head [61] (Figure 5A).

Each organ is covered with sensory hairs, called sensilla, which are innervated by up to four OSNs (Figure 5B). The olfactory sensilla can be divided into three morphological types-basiconic, coeloconic, and trichoid- which differ in size and morphology. Fly OSNs have been studied by single-unit electrophysiology, which is an extracellular recording technique. This method has been used to define the odor responding profiles for every maxillary palp OSN [62] and virtually all of the antennal OSNs [63-66].

Although the morphology of fly olfactory organs looks different from that of the mammalian, the organization of the olfactory system is quite similar to the mammalian olfactory system. Olfactory neurons expressing the same odorant receptor gene converge to a common olfactory glomerulus in the antennal lobe, the functional equivalent of the mammalian olfactory bulb [67, 68].

*Drosophila* larvae also show robust odor-driven behaviors [69, 70]. The paired dorsal organs are known to be the sole olfactory organs in the larva, and only 21 olfactory neurons innervate the dome sensillum in each of the dorsal organs. These OSN axons project to individual glomeruli in the larval antennal lobe [71]. In addition, a functional analysis of the larval-specific receptor repertoire supports a model in which receptors with similar odor specificities send projections to spatially related portions of the antennal lobe to create a distributive spatial map of olfactory information in the larval brain; OSNs that respond to aliphatic compounds project to a central region of the antennal lobe, whereas those that respond to aromatic compounds project more laterally [72].

### 3.3. Olfactory signal transduction in Drosophila (Figure 6)

**Figure 6.**
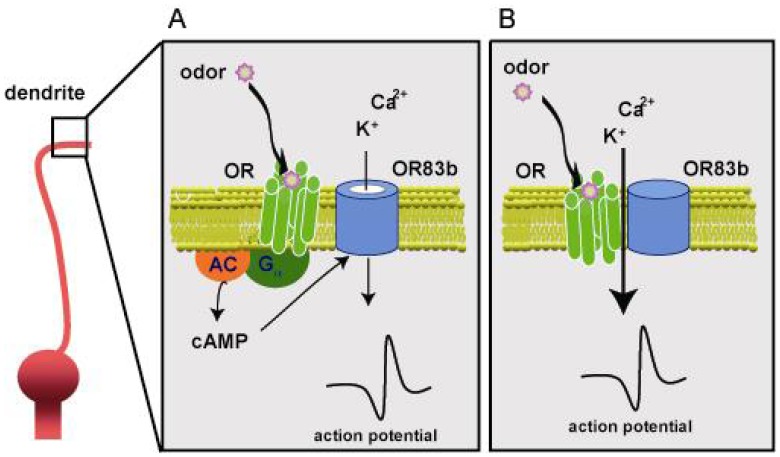
Olfactory signal transduction in *Drosophila*.

Odorant receptor genes, in worm and vertebrate, encode G protein-coupled receptors with seven transmembrane spanning domains, and olfactory signal transduction involves G protein activation and second messenger generation [25, 73] (Figure 6A). However, two recent studies showed that *Or83b*, together with conventional *Or*s, forms a heteromeric ligand-gated cation channel in *Drosophila* [74, 75] (Figure 6B).

There have been several clues that fly olfactory signal transduction may be different from other model organisms. Once fly *Or* genes were identified, a comparison of fly and mammalian *Or* genes revealed that these gene families do not share sequence similarity. Moreover, one member of the receptor family *Or83b* is expressed in virtually all olfactory neurons, and co-expressed with one or a few more odorant receptors [76, 77], whereas there is a strict one neuron to one receptor rule in vertebrates [14, 78]. In addition, the fly odorant receptors appear to have an inverted membrane topology with the N terminus within the cell, opposite to typical G protein-coupled receptors [79].

Taken together, the mechanism of fly signal transduction is still unclear, but may have a distinct odorant signaling (i.e. the odorant receptors are heteromeric ligand-gated cation channels) from the mammalian odorant signaling.

### 3.4. Expression of odorant receptor genes in Drosophila

A large family of seven-transmembrane-domain proteins that encode odorant receptors was identified in *Drosophila* by bioinformatic and molecular approaches [58-60]. The odorant receptor gene family consists of 62 receptor proteins transcribed from 60 *Or* genes by alternative RNA splicing [80]. The fly *Or* genes are widely distributed across all three major chromosomes, but some *Or* genes are found in small clusters. These clusters suggest that some of the ancestral *Or* genes may have undergone recent duplication. However, the *Or* genes are highly diverse, with overall amino acid homology across the family of *Or* genes quite low (only <20% amino acid similarity).

A number of techniques, including *in situ* hybridization, immunohistochemistry, and reporter gene analysis, have been used to examine the expression of individual *Or* genes. These approaches revealed that each gene is expressed in different subpopulations of OSNs either in adult olfactory organs or larval dorsal organs [58-60, 62, 72, 81, 82].

Most *Or* genes are expressed in small subsets of OSNs with the exception of the atypical receptor, *Or83b*. Uniquely among the *Or* genes, *Or83b* is expressed in virtually all OSNs in combination with one or a few conventional ligand-binding odorant receptors [68, 76, 77, 79]. Unlike most other odorant receptors, *Or83b* is highly conserved across insect species [83], and does not directly respond to odorants. Analysis of *Or83b* mutants revealed that *Or83b* is involved in the localization of other odorant receptors to OSN dendrites: In *Or83b* mutants, the conventional odorant receptors are mis-localized to OSN cell bodies rather than dendrites, and animals show severely reduced olfactory responses [77, 79]. In addition, researchers have shown that *Or83b* forms heterodimers with odor receptors using heterologous expression systems [84]. Although its precise mechanism of action is still controversial in certain aspects, two recent studies suggested new insights into the olfactory signaling mechanism via *Or83b* [74, 75] (see below).

### 3.5. Function of odorant receptors in Drosophila

Little knowledge has been concerning the ligand specificity of individual odorant receptors in any species, due to difficulties in expressing odorant receptors in heterologous systems. In *Drosophila*, *Or22a* and *Or22b* were found to be co-expressed in a single class of OSNs on the antenna, and were subsequently characterized in detail by molecular, genetic, and electrophysiological analyses [81]. Molecular analysis mapped both *Or22a* and *Or22b* to the ab3A neurons, and odor responses in the ab3A neuron are completely lost in mutants lacking these receptors. The full odor response spectrum of the ab3A neuron is restored by transgene expression of *Or22a* but not *Or22b*, thus demonstrating that *Or22a* appears to account for the odor response of ab3A [81]. More importantly, a large-scale functional analysis of the fly odorant receptors was accomplished using this deletion mutant as an *in vivo* expression system [62, 65, 72, 85]. This system is based on the ab3A OSN, which in the wild-type antenna responds strongly to several volatile compounds. The endogenous receptors in this OSN, Or22a and Or22b, have been genetically removed, thereby eliminating the normal odorant responses of the OSN. Individual receptors can be expressed in this “empty neuron” via an *Or22a* promoter and the *GAL4*-*UAS* system, and the olfactory responses conferred by the particular receptor can be measured through electrophysiological methods.

Systematic characterization of nearly all the receptors expressed in the antennal basiconic sensilla provided the principle molecular basis of odor coding at the peripheral level [85-87]. Twenty four receptors were decoded in this empty neuron system, and most of them conferred response to food odors. A comparison of the odor response profile of individual odor receptors revealed that many odorant receptors respond to common odors, and thus one odor can activate multiple receptors. Moreover, the odorant receptor determined many odor response properties, including the spontaneous firing rate, signaling mode, and response dynamics of the response, as well as the odor response spectrum of the OSN in which it is expressed [85-87].

In the past few years, there have been particularly intensive studies of the molecular and cellular basis of mate recognition in *Drosophila* [65, 88, 89]. Electrophysiological analysis revealed that the trichoid sensilla, one of the three types of sensilla on the antenna, respond to fly odors. Among all *Or* genes which are expressed in trichoid sensilla, two of them, *Or65a* and *Or67d*, have been shown to respond to cis-vaccenyl acetate (cVA) by using an *in vivo* expression system [65]. cVA is an anti-aphrodisiac pheromone that is produced only by males and which is transferred from males to females during copulation [90]. Different from the case of odorant receptors, cVA does not directly activate receptors but does when cVA is bound to an odorant binding protein, LUSH [91].

When a systematic investigation of the coeloconic sensilla, one of the three major morphological types of olfactory sensilla, was performed through extracellular physiological recordings, researchers found a class of OSNs which are tuned to a small number of specific chemosensory stimuli, such as water vapor, ammonia, and 1,4-diaminobutane, also known as putrescine [66]. No *Or* gene was mapped to these OSNs, and the molecular basis of chemoreception of these stimuli has not been uncovered yet. This suggests that additional classes of chemosensory receptor genes may exist in *Drosophila*.

### 3.6. Carbon dioxide receptors in Drosophila

Many insects respond to CO_2_, including *Aedes* mosquitoes that carry yellow fever and dengue, and *Anopheles* mosquitoes that are responsible for hundreds of millions of cases of malaria each year [92].

One class of olfactory receptor neuron (OSN) in the *Drosophila* antenna, ab1C, has been shown to respond with high sensitivity and specificity to CO_2_ (de Bruyne *et al.* 2001). OSNs of this class send axons to the V glomerulus of the antennal lobe of the brain, which has been implicated in CO_2_ response [93]. There are no *Or* genes that are known to be expressed in this OSN. In *Drosophila*, a highly diverse family of gustatory receptor (*Gr*) genes was identified by bioinformatic and molecular approaches [94]. A total of 60 *Gr* genes compose a superfamily of chemosensory receptor genes together with *Or* genes in *Drosophila* [80]. Two recent studies have shown that two gustatory receptors, *Gr21a* and *Gr63a*, are co-expressed in the neuron that responds to CO_2_, and are sufficient to confer a response to CO_2_ when ectopically expressed in CO_2_-insenstive OSNs [95, 96]. Analysis of ectopic expression in the empty neuron revealed that expression of either receptor alone did not confer a response to CO_2_, but when the two were coexpressed, this produced a physiological response to CO_2_ [96]. *Gr63a* mutant flies lose all electrophysiological and behavioral responses to CO_2_, suggesting that *Gr21a* and *Gr63a* form a heterodimer that acts as a CO_2_ receptor in *Drosophila* [95]; in any case these results suggest the possibility of screening volatile compounds for their inhibition or activation of these proteins. Such compounds could affect the response of insect disease vectors, which are responsible for hundreds of millions of infections each year, to CO_2_ emanations from the human hosts they seek.

*Gr21a* and *Gr63a* are among a small number of *Gr* genes that have orthologs in the malaria vector mosquito *Anopheles gambiae* [97]. The coexpression of *AgGR* genes which are orthologs of *Drosophila* CO_2_ receptors, was sufficient to confer responses to CO_2_ in fly CO_2_-insensitive OSNs [98]. This result may provide a molecular handle to perturb the CO_2_ response in the mosquito, which could have a significant impact on the spread of diseases such as malaria.

## 4. Conclusions

**Figure 7.**
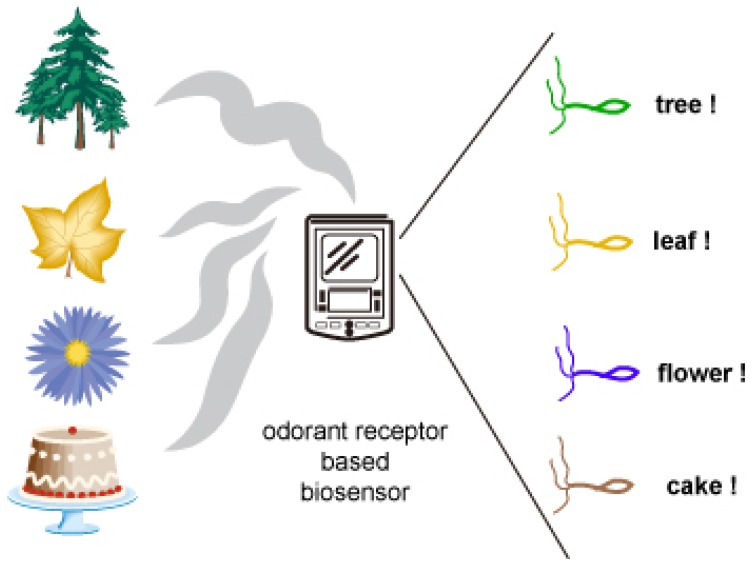
Odorant receptor based biosensor.

Olfaction is an essential sensory modality that influences the quality and the survival of an organism. In the last decade huge progress has been made regarding our understanding of odorant transduction. Challenges still exist, so have technological perspectives been getting brighter. Odorant receptor based biosensors may replace many of the inconvenient and inefficient bioanalytical methods or devices, and ease our life and produce well-being society (Figure 7).
